# Serum Concentrations of Essential Trace and Toxic Elements in Healthy and Disease-Affected Dogs

**DOI:** 10.3390/ani10061052

**Published:** 2020-06-18

**Authors:** Yolanda Cedeño, Marta Miranda, Inmaculada Orjales, Carlos Herrero-Latorre, Maruska Suárez, Diego Luna, Marta López-Alonso

**Affiliations:** 1 Department of Animal Pathology, Faculty of Veterinary, Universidade de Santiago de Compostela, Campus Terra, 27002 Lugo, Spain; yoli19mp@hotmail.com (Y.C.); dluna@doctor.com (D.L.); marta.lopez.alonso@usc.es (M.L.-A.); 2 Faculty of Veterinary Medicine, Universidad Central del Ecuador, Quito EC170521, Ecuador; 3 Department of Anatomy, Animal Production and Clinical Veterinary Sciences, Faculty of Veterinary, Universidade de Santiago de Compostela, Campus Terra, 27002 Lugo, Spain; inma.orjales@gmail.com (I.O.); maruska.suarez@usc.es (M.S.); 4 Rof-Codina Veterinary Teaching Hospital, Faculty of Veterinary, Universidade de Santiago de Compostela, Campus Terra, 27002 Lugo, Spain; 5 Research Institute on Chemical and Biological Analysis, Analytical Chemistry, Nutrition and Bromatology Department, Faculty of Sciences, Universidade de Santiago de Compostela, Campus Terra, 27002 Lugo, Spain; carlos.herrero@usc.es

**Keywords:** trace and toxic elements, serum, dog, inductively coupled plasma-mass spectrometry (ICP-MS)

## Abstract

**Simple Summary:**

Establishing reference ranges for essential and toxic trace elements in dogs is important to assess health status and to enable assessments of the background levels of toxic element exposure. On the other hand, establishing whether trace element concentrations vary in relation to different pathologies is also important. Serum concentrations of trace elements may be effective diagnostic markers and may help in understanding the pathogenesis of different diseases (and the associated causal relationships).

**Abstract:**

This study was designed (i) to establish reference ranges for the essential trace element and background levels of toxic element exposure in the healthy/normal dog population, and (ii) to evaluate whether trace element concentrations vary in dogs suffering from different pathologies. Blood serum samples were collected from 187 healthy and diseased dogs at the Veterinary Teaching Hospital, Faculty of Veterinary Medicine, University of Santiago de Compostela (northwest Spain). The samples were acid digested, and the concentrations of trace elements (Co, Cr, Cu, Fe, Mn, Mo, Ni, Se and Zn) and toxic elements (As, Cd, Hg and Pb) were determined by inductively coupled plasma-mass spectrometry (ICP-MS). This enabled us to establish reference ranges for the essential trace elements and the level of toxic element exposure in dogs, and to identify several clinical situations associated with variations in trace elements in serum. Relative to concentrations in healthy control dogs, statistically significant differences were observed in the concentrations of Cu (significantly higher in hepatic, inflammatory/infectious and oncological categories), Mo (significantly higher in renal category), Se (significantly lower in gastrointestinal category) and Zn (significantly lower in gastrointestinal, inflammatory/infectious and renal categories). Trace element concentrations can be a cause or consequence of disease, and the study findings indicate that trace element determination in serum provides useful information on the pathogenesis of certain diseases. Further research on the serum concentrations of trace elements, particularly in relation to other biochemical parameters and diagnostic tools, may provide valuable information for the diagnosis of diseases in dogs and the disease prognosis.

## 1. Introduction

Good nutrition is essential for preserving the overall health of organisms. Micronutrients, including trace elements, mediate vital biochemical reactions by acting as cofactors for many enzymes, as well as act as centers for stabilizing structures of enzymes and proteins. Although the essentiality of some elements, such as I and Fe, has been discovered two centuries ago, new and vital roles of trace elements in the pathogenesis of regenerative processes, the responses to oxidative stress in the body tissues, and sustaining immunity against pathogens are still been discovered [1,2]. In humans, trace element deficiencies are known to be associated with reduced antioxidant potential in organisms (possibly underlying the onset of cancer and atherosclerosis), accelerated aging, retarded development in growing individuals, increased incidence of abnormal reproduction, immunological abnormalities and lifestyle-related diseases [1,2]. Although information in animals is scarcer, it is expected that trace elements are also involved in the pathogenesis of numerous diseases. For example, Cu [3,4,5,6,7] and other trace elements [8] are known to play a role in chronic hepatitis in dogs, and elevated concentrations of Fe and Cu (capable of inducing oxidative damage) have been detected in the brain tissues of Alzheimer’s patients and in the brains of humans and animals affected by other neurological disorders [9,10]. A recent study in dogs has suggested the role of Mn, Se and Zn in the pathophysiology and/or treatment of epilepsy, and that anti-convulsant therapy may affect Cu and Mo metabolism [11]. On some occasions, the negative effects may also be due to interactions between trace elements and/or trace element imbalances [12]. Trace elements are therefore attracting attention in relation to prophylactic medicine, and diets are formulated to include specific and well-defined trace element concentrations [13].

One of the most difficult problems concerning trace elements, from both nutritional and clinical points of view, is the difficulty in diagnosing trace element disorders, particularly deficiencies. Even in humans, on which much research has been conducted in the last few decades, few methods enable accurate diagnosis, especially in cases of mild to moderate deficiency/excess [1]. Information about trace element concentrations in the blood and tissues of other species, such as dogs, is very limited (mainly related to Cu-associated hepatitis), and reference ranges have not yet been properly established. Although the traditional determination of trace element concentrations in non-invasive samples, such as blood, is very difficult as trace element concentrations are generally very low—making their determination very imprecise, costly and time consuming—the advances in multielement techniques with very low limits of detection, such as inductively coupled plasma-mass spectrometry; ICP-MS, enable the accurate and precise diagnosis of toxic and trace elements in blood by simple, inexpensive methods. This yields detailed information about the levels of trace elements and enables the diagnosis of associated diseases.

In the present study, the serum concentrations of the main trace elements (Co, Cr, Cu, Fe, Mn, Mo, Ni, Se and Zn) and toxic elements (As, Cd, Hg and Pb) were determined in a representative sample (cohort) of dogs in northwest Spain by using a validated ICP-MS method. The first objective of the study was to establish reference ranges of concentrations of the essential trace elements in the healthy/normal dog population and the background level of toxic element exposure in the region. The second objective was to evaluate the potential relationship between serum concentrations of the elements determined and different pathologies in dogs.

## 2. Material and Methods

### 2.1. Animals and Sample Collection

Dogs were treated according to Directive 2010/63/EU on the protection of animals used for scientific purposes and the trial complied with the Spanish legislation on animal care (RD 53/2013, 1 February 2013). The procedures applied were supervised by the Bioethics Committee of the Rof-Codina Veterinary Teaching Hospital, University of Santiago de Compostela (Spain).

Data from a total of 187 dogs attending the Rof-Codina Veterinary Teaching Hospital, Faculty of Veterinary Medicine, University of Santiago de Compostela (northwest Spain), between November 2015 and April 2017 were used in the present study. In all cases, the dogs were considered eligible for study when blood samples were collected for clinical procedures and when a clear and definitive diagnosis was available. A group of 42 healthy animals (both males and females) attending the hospital for castration procedures was considered a control group after clinical examination and pre-operative examination (hematology and basic biochemistry). The other 145 dogs were clinically affected and were classified according to the pathology diagnosed: cardiorespiratory (CR; *n* = 11), dermatological (D; *n* = 11), gastrointestinal (GI; *n* = 24), hepatic (H; *n* = 25), inflammatory-infection (II; *n* = 24), neurological (N; *n* = 13), oncological (O; *n* = 15) and renal (R; *n* = 22) disease. All animals were adult (age range, 1.2 to 12.4 years) and included 52.4% females (*n* = 98) and 47.6% males (*n* = 89). The male/female proportion was similar in all pathological categories, around 50/50.

### 2.2. Sample Preparation and ICP-MS Analysis

Blood samples obtained from the cephalic vein were centrifuged at 3000 rpm for 5 min. Serum was extracted from the samples and stored at −20 °C until analysis. The serum samples were acid digested prior to the determination of trace elements and toxic metals. Briefly, 0.5 mL of serum was mixed with 1 mL concentrated HNO_3_ and 0.5 mL H_2_O_2_ in propylene tubes. The mixture was maintained at 60 °C for 2 h to allow digestion of the samples. The digest thus obtained was diluted by adding 2.5 mL of ultrapure water. The sample solutions were then centrifuged at 2000 rpm for 5 min and the concentrations of trace elements (Co, Cr, Cu, Fe, Mn, Mo, Ni, Se and Zn) and toxic elements (As, Cd, Hg and Pb) were subsequently determined in the supernatant, by ICP-MS (Agilent 7700× ICP-MS system; Agilent Technologies, Tokyo, Japan), according to the analytical procedure described in detail in a previous paper [14].

In order to verify the analytical results obtained, an analytical quality control program was applied. Several analytical blanks (prepared exactly according to the same procedure applied to the serum samples) were included in all batches. The corresponding results were used to calculate the limit of detection (LOD) for each of the elements considered (as 3 times the standard deviation of the blank divided by the slope of the calibration curve). The LOD values obtained were low enough to enable the determination of all elements considered. The accuracy of the method was checked by using certified reference material (CRM) of animal serum NIST-1598a (National Institute of Standards and Technology, Gaithersburg, MA, USA) as well as a set of samples spiked at the appropriate concentration levels (up to 2–10 times higher than the normal levels in the samples). Overall, good recoveries (90–110%) were achieved for both the CRM and the spiked samples. In addition, the intra-sample precision (assessed from 10 repetitions of the same sample) and inter-assay precision (evaluated by preparing 10 digest solutions of the same sample on different days) were also determined, yielding satisfactory values. The results of this quality control program are presented in Table 1.

### 2.3. Data Analysis and Reference Intervals

All statistical analyses were carried out with Statgraphics Centurion XVIII, ver. 18.1.12 (Statistical Graphics, Rockville, MD, USA). The data distribution was checked using the Kolmogorov–Smirnov (K–S) test. The influence of the pathology on trace and toxic elements was evaluated by ANOVA or the Kruskal–Wallis (K–W) test for categories. The correlations between elements were tested by Pearson’s correlation coefficient. All differences were considered significant at *p* < 0.05.

In clinical chemistry, the reference range (or reference interval) for a variable is the range of values of this variable that is deemed normal in healthy animals. The reference range for a particular variable is the interval including the 95% of the values of a reference population and excluding 2.5 % of the values at either end of the range. The limits of this range, i.e., the lower reference limit (LRL) and the upper reference limit (URL), can be estimated by parametric or non-parametric statistical methods for normal (Gaussian) and non-normal distributions, respectively. In the case at hand, after the K–S test, if the variable considered *j* was normally distributed, LRL and URL were calculated as ${\bar{X}}_{j\;}-\;1.96\;SD_{j}$ and ${\bar{X}}_{j\;}+\;1.96\;SD_{j}$, respectively, where ${\bar{X}}_{j\;}$is the mean of the variable *j* and $SD_{j}$ its standard deviation; otherwise, if the variable was not-normally distributed, then LRL and URL were obtained as the 2.5% and 97.5% percentiles [15].

## 3. Results

The reference intervals for essential trace and toxic element concentrations in serum in the healthy dog population in the present study, calculated as indicated in Section 2.3 according to each variable distribution, are shown in Table 2.

**Figure 1:**
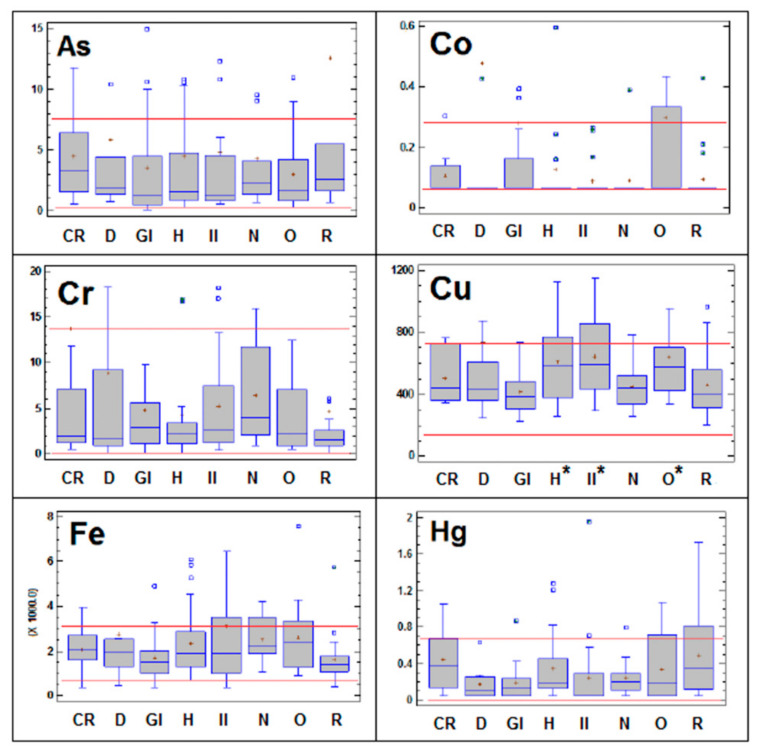

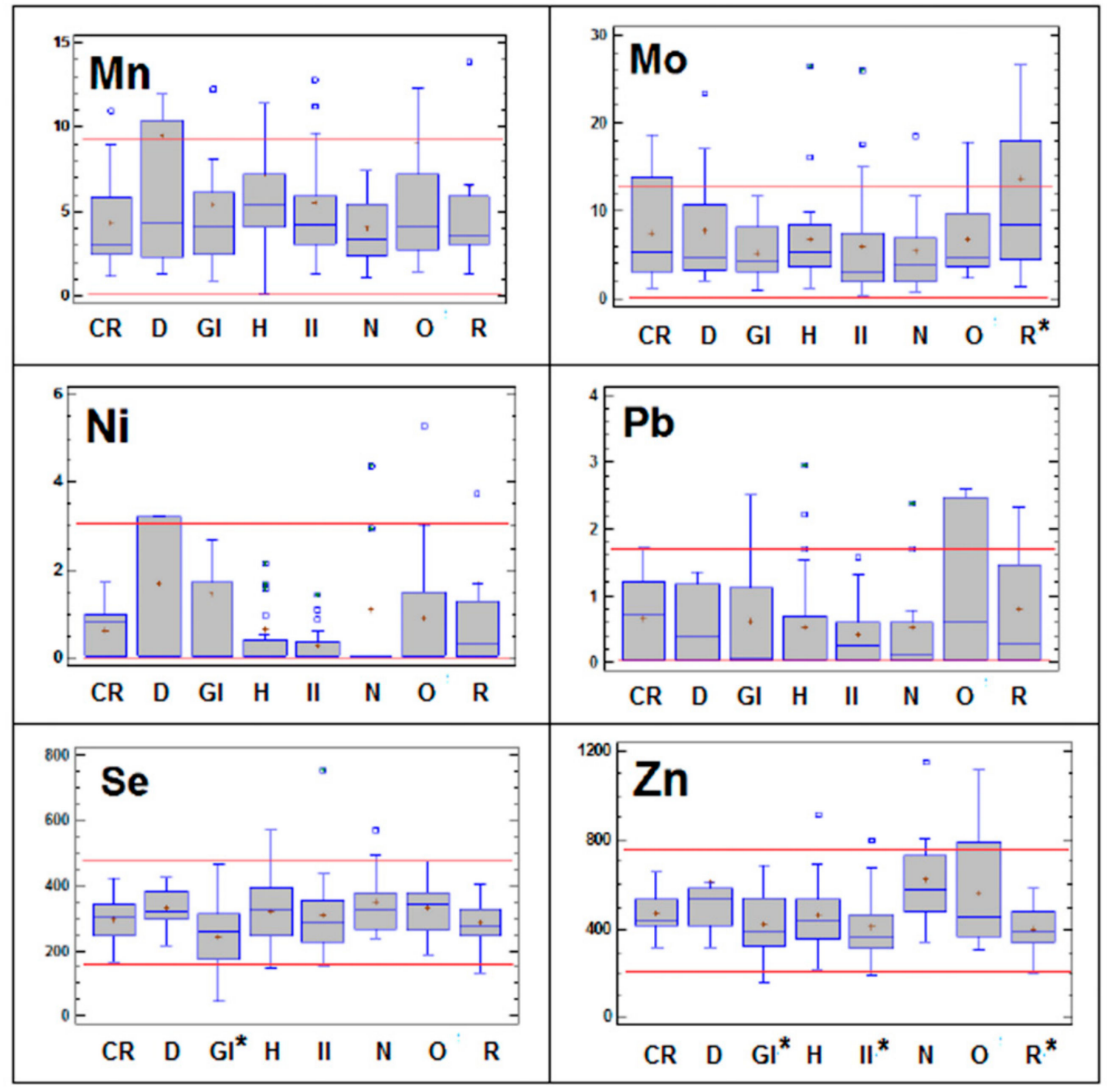
Box-and-whisker plot showing the concentrations of As, Co, Cr, Cu, Fe, Hg, Mn, Mo, Ni, Pb, Se and Zn in the serum of dogs suffering from cardiorespiratory (CR), dermatological (D), gastrointestinal (GI), hepatic (H), inflammatory-infection (II), neurological (N), oncological (O) and renal (R) diseases. All concentrations are expressed in µg L^−1^. The r lines represent the lower reference limit and upper reference limit of the reference interval for each variable obtained from healthy control animals. The horizontal blue line within the box symbolizes the median of the variable; the red cross is the mean value; and the lower and upper boundaries of the box represent the first and third quartiles (thus, the box is the interval covering the middle 50% of the values); whiskers are drawn from the edges of the box to the highest and lowest values (except for values unusually far away from the box). In this case, the outliers, i.e., those points more than 1.5 times the interquartile range (box width) above or below the box, are indicated by blue squares. * Indicates statistically significant difference between the pathological group considered and the control group (*p* < 0.05).

Detailed descriptions of the essential trace and toxic element concentrations in dogs in relation to the pathologies they are suffering are presented in Figure 1. ANOVA revealed statistically significant differences in the essential trace elements for the different groups of pathologies relative to the control group (*p* < 0.05): Cu (significantly higher in hepatic, inflammatory/infectious and oncological categories), Mo (significantly higher in renal category), Se (significantly lower in gastrointestinal illnesses) and Zn (significantly lower in gastrointestinal, inflammatory/infectious and renal categories).

Moreover, detailed analysis of Figure 1 reveals that in the above-described pathologies in which a statistically significant increase in trace elements occurs in the serum (Cu in hepatic, inflammatory/infectious and oncological categories; Mo in renal patients) values were above the upper normal range in more than 25 % of the animals. Although not statistically significant, the same pattern of behavior has been observed for other elements in some pathologies: Co, Fe, Zn, Hg and Pb in oncological disorders; Ni and Mn in dermatological processes; and Fe in individuals suffering from infection/inflammation and neurological diseases.

## 4. Discussion

### 4.1. Reference Intervals of Essential Trace Elements and Levels of Toxic Element Exposure

Information about trace element concentrations in serum or plasma of dogs is very scarce, and reference intervals are not available. As far as we are aware, the only attempt to provide normal or reference ranges in serum of dogs was that made 25 years ago by Puls in a book entitled *Mineral Levels in Animal Health* (1994) [16]. However, the information supplied was limited to the main trace elements, and the absence of knowledge about the origin of these data (including techniques of analysis, limits of detection and other relevant analytical data) prevents its use for comparative purposes. When considering the main essential trace elements, the concentrations of Cu, Fe, Se and Zn in serum from dogs in the present study are reasonably consistent with the normal/adequate range described by Puls [16] in dogs, as well as with other studies published in the literature for this animal species from observational studies (both for the general dog population or healthy control groups; see Table 3).

These values are also consistent with the normal ranges in humans [26] and livestock species, for which more information is available (for a review [16,27]). The results are not surprising, as trace element concentrations in healthy animals receiving adequate diets (most of the control dogs in our study are fed commercial diets fortified with trace elements) are tightly regulated by homeostatic mechanisms. For example, in serum/plasma, 60–70% of Cu is found in ceruloplasmin and corresponds to Cu exported from the liver to tissues; the rest of the Cu is associated with the Cu transport proteins transcuprein (10–30%) and albumin (15–20%), which transport Cu from the intestine to the liver and kidney [5,28]. By contrast, the concentrations of Mn in dogs in the present study are much lower than those considered adequate by Puls [16] (1 order of magnitude) and in other studies in dogs (up to orders of magnitude, see Table 3), but are consistent with those described in studies in dogs [11], humans [26] and ruminants [27] using modern multi-elemental analytical techniques (such as ICP-MS) with very low limits of quantification. The situation for Co is similar, and information in dogs is even scarcer (no data provided by Puls [16]). It should be noted that, the concentrations of both Co (mean: 0.10 µg L^−1^) and Mn (mean: 3.79 µg L^−1^) were much lower in serum than in other tissues and were very close to the limits of quantification of the analytical technique. In this case, conducting an analytical quality control program, including certified reference materials and spiked samples, is essential for guaranteeing good sample analysis. In fact, Co and Mn results in our study were in the same range of the unique study in dogs using ICP-MS analysis (see Table 3) [11].

Precise information about trace elements that are considered only occasional beneficial (Cr, Mo and Ni) is also very limited for all animal species (except Mo in ruminants, because of its particular antagonism with Cu [12]). These elements are only essential at “ultra-trace concentrations”, well below those found in normal diets, and the clinical consequences of their deprivation are only observed when animals receive experimental purified diets [12]. The minimum serum concentrations required to maintain normal metabolism are not well defined but assumed to be well below those in serum. In the present study, Cr, Mo and Ni concentrations are in the low range of those described in other studies (see Table 3).

Finally, considering the toxic elements, the concentrations of As, Hg and Pb were very low (maximum levels of residue 7.89, 0.79, and 1.79 µg L^−1^, respectively), and the concentrations of Cd in all samples were below the limit of detection of the technique. Toxic metals in dogs in the present study are much lower than in other studies in dogs (up to orders of magnitude, see Table 3), and similar results in terms of concentrations have been found in other studies in humans [26] and in cattle from the same geographical region as the present study [29], indicating a low level of environmental exposure in the region.

### 4.2. Essential Trace and Toxic Element Concentrations in Dogs Suffering from Different Pathologies

We are aware that our study involves a reduced number of samples, and consequently our results must be interpreted with caution. However, it is worth noting that the trace element concentrations in serum that are related to pathological disorders in our study have been previously associated to similar disorders, mainly in humans. In some cases, variations in these trace elements have been proposed as markers of disease and indicative of prognosis [30,31]. The most extensive information is available for Cu, and excessive Cu accumulation in the liver is known to occur in dogs suffering from hepatic disorders [3,4,5,6,7,8]. Serum copper concentrations are also higher in human patients suffering from hepatic disorders [31], and elevated Cu:Zn ratios have been proposed as markers of disease in patients with hepatic cirrhosis [31] or hepatocellular carcinoma [32]. The role of Cu in the acute-phase protein ceruloplasmin in controlling acute inflammatory-infectious disorders, leading to a marked increase in Cu serum concentrations, is well known in both dogs [22,33] and humans [34]. There is evidence of increased concentrations of Cu and decreased concentrations of Fe and Zn in serum of dogs infected with *Hepatozoon canis* [22] and with *Rangelia vitalii* (Apicomplexa: Piroplasmorida) [33]. The concentrations of Cu in human serum are increased in various carcinomas [32,35]. Similarly, in a recent study, increased liver Cu and decreased Fe, Se and Zn levels were observed in the livers of dogs with hepatocellular carcinoma [36]. Changes in Cu and Zn serum and tissue levels have been observed in both human and animal models with neoplasms [31,32,35,36], and it has not yet been established whether altered Cu and Zn concentrations are the cause or the effect of the malignancy. Hence, the usefulness of serum Zn and Cu determinations for cancer prevention, detection, monitoring treatment and prognosis requires further investigation.

A large variety of inflammatory gastrointestinal diseases in dogs are associated with altered Zn metabolism or deficiency, and decreased levels of Zn in the blood of dogs with diarrhea have been reported [37]. Acute and chronic diarrheal disorders may cause Zn deficiency because of increased loss or decreased absorption of the element or altered immunity. When the small intestinal barrier is altered by inflammation, Zn supplementation may help correct the deficiency and also improve the small bowel mucosal capacity to absorb water and electrolytes [38]. Chronic deficiency of Zn increases inflammation, and several pathologies are thus characterized by imbalanced Zn homeostasis [2].

In addition, serum Zn concentrations are low in human patients with some renal clinical disorders, including nephrotic syndrome and renal insufficiency associated with dialysis [30]; urinary Zn excretion increases and symptoms of Zn deficiency are common in these patients. It is not clear whether these disease states are indicative of true symptomatic or asymptomatic Zn deficiency or merely reflect a decrease in available Zn binding proteins. Low serum Zn concentrations and high urinary Zn excretion in patients with nephrotic syndrome do not appear to be due to loss of Zn bound to urinary proteins.

On the contrary, Mo concentrations in serum increase in dogs suffering from renal disorders. This could be related to the ratio of renal excretion since it is their main excretion route. In a recent study in dogs and cats with chronic interstitial nephritis was observed that the ratio of Mo excretion in urine was lower than in healthy animals [39]. In humans, it has been suggested that high serum Mo concentrations could contribute to dialysis-related bone disease in patients requiring long term hemodialysis, as massive Mo accumulation causes joint deformity and arthritis [40].

Selenium deficiency is also known to occur in patients with severe gastrointestinal disorders. Selenium deficiency is mainly related to malabsorption, and low Se levels are almost invariably present in human patients requiring parenteral supplementation due to gut failure [41]. Oxidative stress has been implicated in the pathogenesis of gastrointestinal disease in dogs [37], as it enhances the need for Se and further aggravates the deficiency.

Moreover, many observational studies in human patients have detected a relationship between tumor development and elevated serum concentrations of Co [42,43,44] and Fe [45,46]. However, evidence for the carcinogenetic potential of Hg or Pb is scarce. As Pb was highly correlated with Cu (r = 0.89, *p* < 0.001) and Hg with Fe (r = 0.69, *p* < 0.001) in the present study, it is possible that exposure to these metals may have occurred simultaneously in the individuals under study. The presence of high Zn serum levels in the individuals suffering oncological disorders was unexpected as elevated Zn concentrations in serum have not been associated with cancer in previous studies and are even considered a protective factor against tumor development [47,48]. Interestingly, Ni and Mn concentrations above the upper limit in serum were observed in a high proportion of dogs suffering from dermatological disorders. In humans, contact dermatitis associated with Ni is well documented [49,50] and Mn allergic contact dermatitis has also been reported [51,52]. Finally, the Fe concentrations above the normal range in a large proportion of individuals suffering from infection/inflammation and neurological diseases is noteworthy, and the risk to these patients is well documented. Clinical conditions associated with excess Fe in the host may increase the risk for infection, as bacteria require Fe to enable them to multiply in the host, and microbiological studies show a close relationship between Fe availability and bacterial virulence. In human and animal studies, the administration of parenteral Fe during infection has already been shown to be harmful [53]. In the brain, Fe is involved in many fundamental biological processes, including oxygen transport, DNA synthesis, mitochondrial respiration, myelin synthesis, and neurotransmitter synthesis and metabolism. Iron homoeostasis is required to maintain normal physiological brain function, whereas faulty regulation of Fe homoeostasis can cause neurotoxicity. A recent study regarding serum concentrations of trace elements in epileptic and healthy dogs described higher Fe levels in untreated epileptics dogs compared to healthy and epileptic dogs with treatment [11]. When Fe concentrations exceed the cellular Fe sequestration capacity of storage proteins or other molecules, the concentration of Fe in the labile Fe pool may increase, with possibly harmful effects [10,54].

## 5. Conclusions

Information on the main essential trace and toxic elements in the serum of a representative cohort-sample of dogs (in NW Spain) was used to establish reference ranges for the essential trace elements and levels of toxic element exposure. In addition, several clinical situations associated with variations in trace elements in serum were identified. In most cases, the patterns of these variations are consistent with those previously described in studies on elemental serum levels in humans. Our results, although preliminary, indicate that trace element determination in serum could provide useful information about the pathogenesis of certain diseases (and about the associated causal relationships). Further investigation of trace element concentrations in serum, together with other biochemical parameters and diagnostic tools, may provide valuable information regarding the diagnosis of disease and prognosis in dogs.

## Figures and Tables

**Table 1:** Results of the analytical quality program applied for the ICP-MS determination of the essential trace and toxic elements in serum of dogs in the present study.

Metal	Detection Limit	Animal Serum SRM1598a		Spiked Samples
	(µg L^−1^)	Certified Value * (µg L^−1^)	Recovery (%)	Recovery * (%)
**As**	0.039	(0.3)	86.1 ± 6.9	93.8 ± 7.4
**Cd**	0.014	0.048 ± 0.004	ND	106 ± 8
**Co**	0.027	1.24 ± 0.07	93.3 ± 5.6	95.1 ± 6.7
**Cr**	0.025	0.33 ± 0.08	91.2 ± 5.1	91.8 ± 4.4
**Cu**	0.051	1580 ± 90	92.5 ± 2.5	104 ± 2
**Fe**	0.053	1680 ± 60	102 ± 8	106 ± 10
**Hg**	0.019	0.32 ± 0.19	94.1 ± 4.7	89.1 ± 8.4
**Mn**	0.026	1.78 ± 0.33	112 ± 13	100 ± 7
**Mo**	0.011	5.5 ± 1.0	98.3 ± 3.7	95.7 ± 6.0
**Ni**	0.024	0.94 ± 0.18	97.5 ± 4.8	103 ± 7
**Pb**	0.015	-		102 ± 7
**Se**	0.080	134.4 ± 5.8	97.8 ± 2.3	99.1 ± 7.7
**Zn**	0.620	880 ± 24	95.8 ± 3.1	101 ± 8

* Expressed as mean ± SD. In brackets only indicative values. ND: not detected. SRM: standard reference material.

**Table 2:** Descriptive statistic for trace and toxic element concentrations in serum of the healthy/normal dog samples (*n* = 42) and calculated reference intervals. (All results are in µg L^−1^).

Metal	Mean	SD	Median	Normal Distribution (K–S Test)	Reference Interval	
					Lower Reference Limit	Upper Reference Limit
**As**	1.86	1.44	1.36	NO	0.22	7.89
**Cd**	ND	-	-	-	-	-
**Co**	0.10	0.07	0.07	NO	0.07	0.28
**Cr**	2.73	2.54	2.17	NO	0.63	13.7
**Cu**	422	148	377	YES	132	712
**Fe**	1938	639	1939	YES	686	3190
**Hg**	0.24	0.22	0.16	YES	0.0	0.67
**Mn**	3.79	1.86	3.47	YES	0.14	7.44
**Mo**	5.48	3.36	4.35	YES	0.00	12.1
**Ni**	0.72	0.94	0.06	NO	0.06	3.01
**Pb**	0.55	0.60	0.30	YES	0.0	1.73
**Se**	315	82	310	YES	154	447
**Zn**	489	143	464	YES	209	769

ND: not detected. SD: standard deviation. K-S: Kolmogorov–Smirnov.

**Table 3:** Serum concentrations of essential trace and toxic elements (expressed in µg L^−1^ and arithmetic mean values) in observational studies in dogs.

Type Study/Country	*N*	Element													References [16]
		As	Cd	Co	Cu	Cr	Fe	Mn	Hg	Mo	Ni	Pb	Se	Zn	
Reference Values ^1^			3–5 ^a^		200–800		940–1220	20			1.8–4.2	10–100 ^a^	220 ^a^	700–2000	
*General Dog Population*															
Turkey ^1^	*73*			24	830		1320	10			13	100		730	[17]
Poland ^2,b^	*48*	556			1363	249	1690	683				489		1523	[18]
Italy ^2,c^	*31*	390			780	130		20	510		230	60	230	1970	[19]
*Control Healthy Dogs*															
India ^1^	*10*			36.6 ± 3.6	1050 ± 21		872 ± 21	172 ± 2						642 ± 10	[20]
USA ^3,d^	*50*			0.30	450		1625	3.15		8.45			300	740	[11]
Bulgaria ^2^	*10*				946 ± 143			24 ± 5					291 ± 57	1692 ± 180	[21]
Turkey ^4^	*10*				1006 ± 12		860 ± 199							620 ± 76	[22]
USA ^1,4^	*50*					4.66 ± 2.83	1751 ± 567							1220 ± 360	[23]
Turkey ^4,b^	*16*				511 ± 59		1543 ± 240	20 ± 1							[24]
Iran ^4^	*14*				1514 ± 255		1730							593 ± 226	[25]

^a^ Whole blood, ^b^ urban area, ^c^ polluted area, ^d^ median. Analytical techniques: ^1^ atomic absorption spectrophotometer (AAS); ^2^ inductively coupled plasma optical emission spectroscopy (ICP-OES); ^3^ inductively coupled plasma mass spectroscopy (ICP-MS); ^4^ UV spectrophotometry.
